# NSCLC: from tumorigenesis, immune checkpoint misuse to current and future targeted therapy

**DOI:** 10.3389/fimmu.2024.1342086

**Published:** 2024-02-07

**Authors:** Leona Raskova Kafkova, Joanna M. Mierzwicka, Prosenjit Chakraborty, Petr Jakubec, Ondrej Fischer, Jozef Skarda, Petr Maly, Milan Raska

**Affiliations:** ^1^ Department of Immunology, Faculty of Medicine and Dentistry, Palacky University Olomouc, Olomouc, Czechia; ^2^ Department of Immunology, University Hospital Olomouc, Olomouc, Czechia; ^3^ Laboratory of Ligand Engineering, Institute of Biotechnology of the Czech Academy of Sciences, Vestec, Czechia; ^4^ Department of Respiratory Diseases and Tuberculosis, University Hospital Olomouc, Olomouc, Czechia; ^5^ Institute of Clinical and Molecular Pathology, Faculty of Medicine and Dentistry, Palacky University Olomouc, Olomouc, Czechia; ^6^ Department of Pathology, University Hospital Ostrava and Faculty of Medicine, University of Ostrava, Ostrava, Czechia

**Keywords:** PD-1, PD-L1, immune checkpoint, NSCLC, CTLA-4, targeted therapy

## Abstract

Non-small cell lung cancer (NSCLC) is largely promoted by a multistep tumorigenesis process involving various genetic and epigenetic alterations, which essentially contribute to the high incidence of mortality among patients with NSCLC. Clinical observations revealed that NSCLC also co-opts a multifaceted immune checkpoint dysregulation as an important driving factor in NSCLC progression and development. For example, a deregulated PI3K/AKT/mTOR pathway has been noticed in 50-70% of NSCLC cases, primarily modulated by mutations in key oncogenes such as ALK, EGFR, KRAS, and others. Additionally, genetic association studies containing patient-specific factors and local reimbursement criteria expose/reveal mutations in EGFR/ALK/ROS/BRAF/KRAS/PD-L1 proteins to determine the suitability of available immunotherapy or tyrosine kinase inhibitor therapy. Thus, the expression of such checkpoints on tumors and immune cells is pivotal in understanding the therapeutic efficacy and has been extensively studied for NSCLC treatments. Therefore, this review summarizes current knowledge in NSCLC tumorigenesis, focusing on its genetic and epigenetic intricacies, immune checkpoint dysregulation, and the evolving landscape of targeted therapies. In the context of current and future therapies, we emphasize the significance of antibodies targeting PD-1/PD-L1 and CTLA-4 interactions as the primary therapeutic strategy for immune system reactivation in NSCLC. Other approaches involving the promising potential of nanobodies, probodies, affibodies, and DARPINs targeting immune checkpoints are also described; these are under active research or clinical trials to mediate immune regulation and reduce cancer progression. This comprehensive review underscores the multifaceted nature, current state and future directions of NSCLC research and treatment.

## Introduction

Lung cancer is one of the most frequently diagnosed tumors (2023: 12.5% of all cancers) and the most common cause of death in cancer diagnoses worldwide (2023: 21% of the total number of cancer deaths) (1, 2). It is the second most prevalent cancer in women and the most frequent cancer in men worldwide.

Lung cancer development is a heterogeneous and multi-step process that involves several genetic and epigenetic alterations, which are necessary for tumor cell turnover and evasion of the immune system surveillance allowing cancer progression and metastasis. Non-small cell lung cancer (NSCLC) is one of the two main types of lung cancers and covers approximately 80–85% of all lung cancer cases. The NSCLC could be divided into squamous cell carcinoma, adenocarcinoma, and large cell carcinoma, and rare types such as adenosqamous carcinoma, large cell neuroendocrine carcinoma, etc. (3), based on the type of tumor cells and typical histological features. This review focuses on immune checkpoint disability participating in the NSCLC development and immune checkpoint inhibitors (ICI) therapy.

### Clinical aspects of NSCLC

Early diagnosis of lung cancer is difficult since symptoms are not specific and can be present in a wide range of other disease conditions. The early stages are often asymptomatic, so lung cancer is frequently diagnosed in its advanced stages, commonly associated with metastases. Adenocarcinoma tends to be found earlier. NSCLC metastases are unfavorable prognostic factors associated with shorter survival. Approximately 30–40% of patients are diagnosed with metastatic stage of NSCLC (4, 5). Disseminating tumor cells (DTCs) (6) escape immune elimination mechanisms and spread via the lymphatic system or the bloodstream (7). To survive in the circulation, DTCs could escape immune elimination (e.g. by NK cells) using a protective ‘platelet coat’ formed from adherent activated platelets (8). Typically, bones are detected as common sites of NSCLC metastases, followed by the brain, liver and adrenal glands. 25-40% of NSCLC patients develop brain metastases during the disease within the first 2 years after diagnosis of the primary tumor (9, 10). Liver metastases at the time of diagnosis or before starting chemotherapy are associated with a poor prognosis. The liver is a highly immunosuppressive environment, which can tolerate the presence of antigens without activating the immune response. This could lead to ineffective local responses to immunotherapy (11). Thus, different metastatic sites (organs) may exhibit different responses to ICI therapy due to other immune signaling within the tumor microenvironment (TME) (12).

According to the 7^th^ edition of TNM classification of lung cancers issued by the American Joint Committee on Cancer (AJCC) (13), different stages of NSCLC can be defined in terms of TNM staging system, assessing primary tumor size (marked as T1 to T4), the propagation of tumor into lymph nodes (N0-N3) and the presence (M1) or absence (M0) of metastasis. The TNM 9^th^ edition is the current classification of lung cancer and is crucial for the choice of therapeutic approaches and is of prognostic significance (14). The treatment of lung cancer depends on the histological type, stage, condition and comorbidities of the patient, as well as on predictive markers and tumor genetics (15). Stages I-IIIA are considered operable. From stage IIA on, adjuvant chemotherapy is indicated. In the presence of risk factors, adjuvant chemotherapy is also indicated for stage IB. Inoperable cases are treated with radiotherapy and from stage IIA onwards, chemoradiotherapy is recommended, both sequentially and concomitantly. Stage III is a very heterogeneous group of patients (16). These patients should be assessed by an interdisciplinary indication board. For example, in operable patients, a decision about the choice of therapy has to be made based on the patient’s clinical situation, organ functions, other illnesses and the location/extent of the disease. Primary surgical resection is indicated when possible and safe, either with or without adjuvant chemotherapy. Certain subsets of patients benefit from adjuvant tyrosine kinase inhibitors (TKIs) therapy (osimertinib in EGFR-mutated NSCLC (17)) or alectinib in ALK-mutated NSCLC (18), or adjuvant immunotherapy with immune-checkpoint inhibitors (ICI). In case of extensive N2 noda involvement, an adjuvant radiotherapy is considered (19). Recent studies also show potentially large benefits of neoadjuvant chemoimmunotherapy, sometimes supplemented with adjuvant therapy (perioperative therapy). The inoperable stage III is treated with concomitant or sequential chemoradiotherapy (20), or palliatively with stage IV regimens (when radiotherapy is not possible). Therapy with ICI became a real breakthrough in the field of stage IV treatment since a group of patients, receiving this treatment survived for a long time, and some of them reached permanent remission (21, 22). Deciphering the complexity of immune response to tumor cells may lead to a more detailed characterization of mechanisms that apply to initial or secondary tumor resistance to ICI, which will allow future, more accurate identification of patients potentially benefiting from ICI treatment and the development of new drugs blocking the emergence of secondary or even primary tumor resistance.

### Genomic aberration in NSCLC

Genomic instability is a hallmark of all cancers. Acquired genetic alterations are tightly associated with an increased proliferation rate based on the mutation burden of tumor suppressors and oncogenes involved in signaling pathways regulating cell division. The PI3K/AKT/mTOR pathway is deregulated in 50-70% of NSCLC (23, 24) by a *variety* of mechanisms including activating mutations in Anaplastic lymphoma kinase (*ALK*), Epidermal growth factor receptor (*EGFR*), Kirsten rat sarcoma viral oncogene homolog (*KRAS*), Phosphoinositide 3-kinase (*PI3K*), or protein kinase B (*AKT*) (24, 25), by *PIK3CA* amplification, or by the loss of negative regulation through the tumor suppressor gene *PTEN* (26).

The common oncogenic driver in NSCLC is *EGFR* proto-oncogene (27–29). Patients diagnosed with classical EGFR mutations, i.e., L858R or exon 19 deletions (Ex19del), were noticed for substantial improvements in clinical outcomes after treatment with first-, second-, or third-generation of TKIs (29, 30). Genetic mutations and their overexpression in *EGFR* often result in constitutive activation of tyrosine kinases (30, 31), which causes deregulation of PI3K/AKT/mTOR, RAS/RAF/MEK/ERK and JAK/STAT signaling pathways. Recent studies have also associated the EGFR expression in NSCLC with frequent lymph node metastasis, poor chemosensitivity (32, 33) and reduced survival rate (25, 30, 31).

Tumor suppressor genes (TSG), that are regularly inactivated in NSCLC, are tumor protein p53 (*TP53)*, cyclin-dependent kinase inhibitor 2A (*CDKN2A*), fragile histidine triad protein (*FHIT*), Ras association domain family protein1 isoform A (*RASSF1A*), Phosphatase and Tensin Homolog (*PTEN*), retinoblastoma 1 (*RB1*), and serine-threonine kinase 11 (*STK11*) (25). Among these, *TP53* has most often been noted for carrying substantial genetic alterations, i.e., hemizygous deletion of 17p13, occurring in 65% of NSCLCs (34). *RB1*, the first TSG described in lung cancer (35), is deactivated in 10-15% of NSCLC. ALK was initially identified as a fusion protein EML4-ALK (36, 37), present as a rearrangement mutation in 5% of patients with NSCLC (38), and was marked as a driver tumor mutation in NSCLC. Patients diagnosed with ALK fusion protein, which is constitutively active, are highly sensitive to ALK inhibitors such as Alectinib, Ceritinib, and Crizotinib (39). Moreover, ALK was reported to cause the activation of multiple signaling pathways, including PI3K/AKT, JAK/STAT, MEKK2/3, and MAPK pathways leading to enhanced proliferation (40). Increased proliferation rate is not only based on the accumulation of genomic mutations but is also related to increased telomerase activity, which is augmented in 80% of NSCLCs. Telomerases promote the progression of tumors by maintaining the telomeres above a critically short length, which results in apoptosis inhibition. The development and progression of NSCLC are further connected with aberrant DNA methylation, which can epigenetically modify gene expression (41). A combination of methylation loci can be taken as a prognostic signature for NSCLC. For instance, 17 methylation loci are identified in 13 genes, which were previously reported to be involved in lung cancer tumorigenesis (42).

The tumor mutation burden (TMB) is the number of genetic mutations found in the DNA of cancer cells. It is known that the number of mutations can vary across different tumor types. Tumors with a high mutation burden have the potential to generate a larger number of neoantigens, making them more immunogenic TMB numbers may be a useful biomarker for immunotherapy treatments for advanced cancer patients. However, TMB testing is problematic for several reasons: insufficient studies verifying tumors for which this testing is useful; TMB screening is expensive, making this screening unavailable to most patients; and tumors with low TMB may develop high TMB in response to the used chemotherapy (43).

### Immune system and its role in NSCLC

#### Activation of the immune system by tumor cells

Tumor cells are recognized by T cells (44–46) through tumor-specific antigens (TSA) and tumor-associated antigens (TAA) (47, 48). TSAs are neoantigens, that are entirely absent in normal tissues and thus not tolerated by the immune system. Karasaki et al. (49) found in a group of NSCLC patients that a median of 46 potential neoantigens (pNeoAgs) are generated per one adenocarcinoma patient and 95.5 pNeoAgs per one squamous cell carcinoma (SCC) patient (50). Selection pressures from a diverse TME further affect neoantigen presentation, allowing immune escape and the clinical progression in NSCLC (50, 51). In contrast, TAAs refer to antigen molecules present on both tumor and some normal cells at various stages of development, maturation, or activation. In general, tumor cells express higher TAA levels in comparison to most normal tissues. The most frequently recognized TAAs in NSCLC patients are Aurora kinase A, p53, HER2/neu, and NY-ESO-1. DESTINY-Lung02 demonstrated deep and durable antitumor responses of Trastuzumab deruxtecan in patients with mutated *HER2* NSCLC (52).

Previous studies have shown that T cell responses against one or more of these most common TAAs have significantly improved relapse-free survival in NSCLC patients (46), which could be attributed to tumor cell apoptosis induced by cytotoxic T lymphocyte (CTL), a main subset of tumor-infiltrating lymphocytes (TILs) (53). The TILs count was considerably linked with improved survival as well as tumor grade, size, vascular invasion, and stage of tumor differentiation in NSCLC (54). Additionally, the presence of TILs in the early of NSCLC was linked with an improvement in survival rate and reduced risk of systemic recurrence (55).

**Figure 1:**
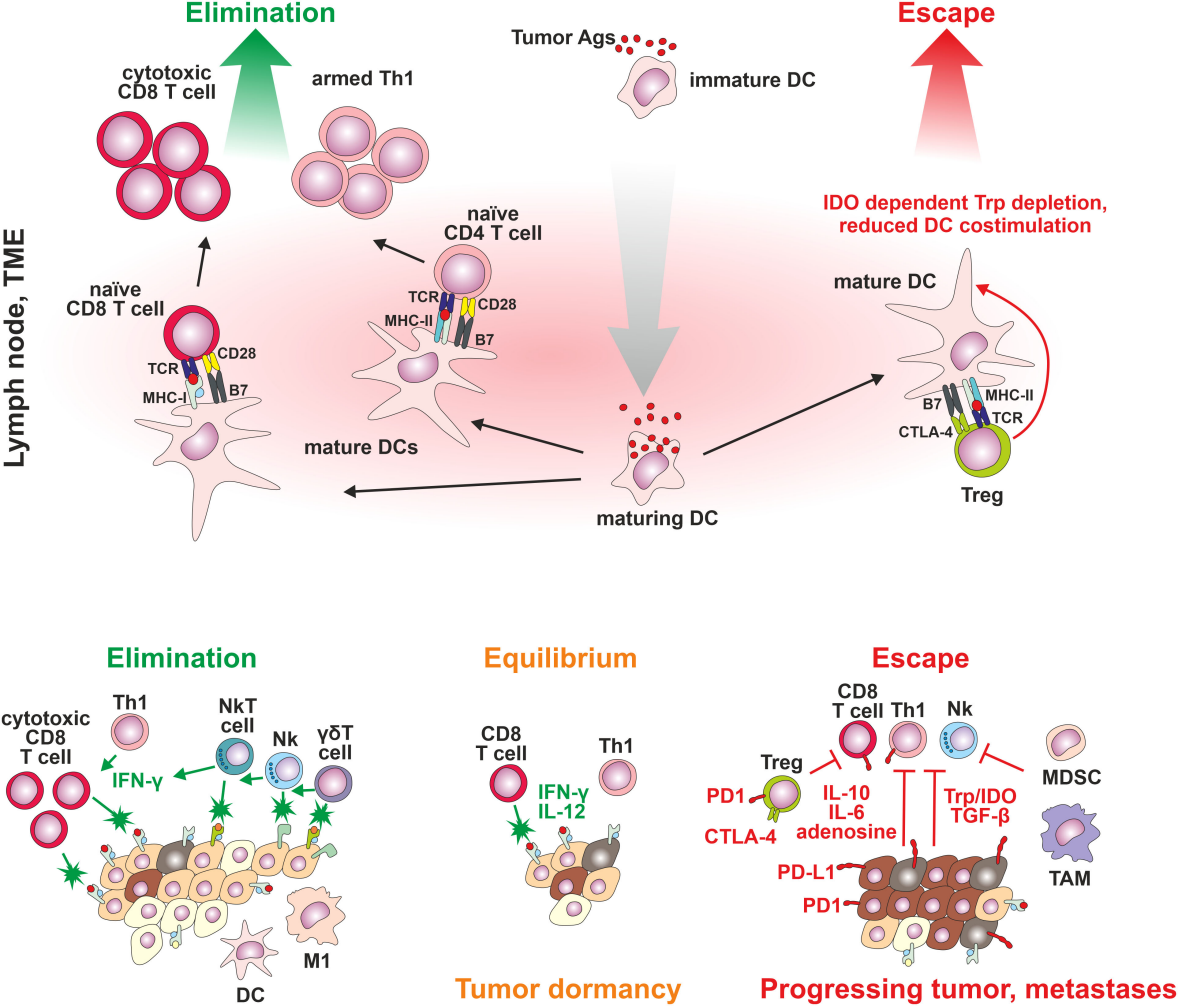
The cancer immunoediting concept. Three steps of cancer development: Stage 1: elimination: control of tumor cell proliferation associated with activation of innate and tumor-specific immune responses – immune system eliminates cancer cells; Stage 2: equilibrium: tumor cell variants may not be completely eliminated but *antigen-specific immune mechanisms effectively suppress cancer growth*; and Stage 3: escape: high genetic instability results in the emergence of proliferating tumor cells poorly recognized by the immune system, not responding to immune-mediated cell-elimination mechanisms, or establishing an immunosuppressive tumor microenvironment.

Recognition of tumor cells by the synergistic action of tumor antigen-specific and innate immunity could induce either complete or partial tumor elimination, with some tumor cells becoming dormant. The tumor dormancy is described as the equilibrium phase during which tumor cell outgrowth is inhibited by the activity of T cells, IL-12 and IFN-γ, indicating the dominant contribution of adaptive immunity. The tumor cells could remain in the equilibrium phase through the rest of the subject’s life. Nevertheless, constant immune pressure, particularly on genetically unstable tumor cells, could lead to the emergence of tumor cells, which are no longer controllable by the immune system, leading to the tumor escape phase with clinically manifested disease. The tumor cells escape due to either a) the loss of TAA or TSA expression or by deviations in antigen processing or presentation by tumor cells, b) the loss of sensitivity of tumor cells to immune-induced cell-elimination mechanisms, including overexpression of antiapoptotic proteins BCL2 and STAT3, or c) immunosuppressive action of TME (56). The suppression of the immune system could be mediated within the TME by a) inhibitory action of tumor and stromal cell-surface expressed receptors (immune checkpoints such as PD-1/PD-L1, suppressive major histocompatibility molecules HLA-E/G, ectoenzymes CD39/73, thrombospondin receptor (CD47), IL-2 receptor β subunit (CD122), or CD155) or by secreted factors, such as interleukin-10 (IL-10), and transforming growth factor-β (TGF-β), or b) by tumor-mediated attraction of immunosuppressive cell populations such as Treg cells, myeloid-derived suppressor cells (MDSC) of either monocytic or polymorphonuclear lineage, tumor-associated macrophages (TAMs), or tumor-associated neutrophils (TANs). Such immune cells could be attracted by a disturbance in the expression of many cytokines, chemokines and adhesive molecules within the TME (57–59). Treg cells, as one of the most discussed regulatory/immunosuppressive populations within TME, can inhibit the proliferation and activity of tumor-specific Th cells and CTL and NK cells by secretion of transforming growth factor-β (TGF-β), or immunosuppressive cytokines IL-10 and IL-35 and by surface expression of IL-2 receptor subunit α (CD25) (60). Treg cells could also modulate the activity of dendritic cells (61). The count of Treg cells within the tumor (62) can be used as an independent biomarker to predict the survival rate in NSCLC patients (63). Nevertheless, the Treg cells within a tumor consist of very heterogeneous populations of either phenotypically stable suppressive/regulatory Tregs (forkhead transcription factor 3 - FoxP3^++^) and phenotypically unstable cells (FoxP3^+/-^), which could, under specific TME environment, switch their phenotype to effector Th1, Th2, Th17, or Tfh cells contributing to tumor elimination. In general, TME Tregs are highly activated, and express high levels of inhibitory immune checkpoint ligands such as PD-1, cytotoxic T lymphocyte-associated protein 4 (CTLA-4), lymphocyte activation gene-3 (LAG-3) and T cell immunoreceptor with Ig and ITIM domains (TIGIT), and also co-stimulatory molecules such as CD27, ICOS, OX40, 41BB, and GITR (64, 65). From a diagnostic point of view, the composition of Treg cell subsets in the blood, tissue, and tumor is distinct (66) (**Figure 1**).

TAM and tumor stromal cells have been suggested to release IL-10 and TGF-β. Furthermore, tumors and other cells present in the TME (dendritic cells, macrophages or endothelial cells) increase expression of the indoleamine-2,3-dioxygenase (IDO) – an immunosuppressive enzyme that cause a decrease in tryptophan amino acid concentration in extracellular environment (essentially required for the T cell function), induce the tumor-specific T cells exhaustion, and promote activation of Treg populations (67).

#### Immune cell composition and localization in primary NSCLC tumor

Spatial tumor heterogeneity occurs in most tumor types, including NSCLC, at both genetic and immunological levels, leading to a heterogeneous immune response toward individual cancer cells. This may be one of the main reasons for resistance development in cancer against immunotherapy, as it is very difficult to evaluate the actual status of immune response from available tumor tissue samples. For example, assessing the immune status in the center and periphery of the tumor as well as in distinct parts of the tumor can give completely different results. Furthermore, at different time points during various phases of therapy, temporal tumor heterogeneity plays an important role. Considering the findings mentioned above, small biopsies can give false results compared to surgically resected specimens.

NSCLC tumors can contain a high number of immune cells, including B cells, macrophages, Th cells, CTL, dendritic cells, NK cells, neutrophils, basophils, eosinophils, mast cells, myeloid-derived suppressor cells (MDSC), tumor-associated neutrophils (TANs), tumor-associated macrophages (TAMs) and other. 47% of all leukocytes, characterized by surface expression of CD45^+^, are represented by T cells, of which 26% are CD4^+^ T cells, followed by 22% of CD8^+^ T cells (68, 69). CD8^+^ T lymphocyte population present in NSCLC tumors has heterogeneous PD-1 expression levels. The most important CD8^+^ cells are those with high PD-1 expression since they show a high tumor recognition capacity which recruits the immune cells to tertiary lymphoid structures (TLS). The presence of PD-1 high CD8^+^ T cells was reported to be associated with favorable response and survival of NSCLC patients treated with PD-1 inhibitors (70). The percentage of B cells varies from 4.4 to 15.9% between studies (69, 71). This significant variance in B cell proportions has been suggested due to different tumor tissue sampling, while B cells reside primarily at the periphery of the tumor tissue, where they cluster with DCs and T cells (69). DCs represent 2.1% of all immune cells in NSCLC tumors of which plasmocytoid DC (pDC; HLA-DR^+^CD123^+^) represent 1.2% of all leukocytes, myeloid DC (mDC2; HLA-DR^+^CD11c^+^CD1c^+^) represent 0.8% and CD141^+^mDC (mDC1; HLA-DR^+^CD11c^+^CD141^+^) represent 0.1% of all leukocytes (72). mDCs present antigen to naïve T cells and contribute to CTL generation, pDCs produce type I IFNs, thus enhancing the antitumor immunity (73). Macrophages represent 4.7% and NK cells represent 4.5% of the immune cells in NSCL. Granulocytes are represented mainly by neutrophils (8.6%), followed by mast cells (1.4%), basophils (0.4%) and eosinophils (0.3%) (69).

Intratumor immune cells are able to form tertiary lymphoid structures (TLSs) placed at the periphery of tumor tissue (74). TLSs comprise a B cell area, neighboring a T cell area covering clusters of mature DCs and T cells (71, 75). Previous studies have established the TLSs as predictive biomarkers for the efficacy of immunotherapy in sarcoma, melanoma, and renal cell carcinoma (76, 77). Their critical role as a biomarker in NSCLC is under investigation (78, 79).

### Tumor-triggered immune cell suppression

Lungs are potent immune organs and contain macrophages, DCs, NK cells and adaptive immune cells (T and B cells). Therefore, immune checkpoints in NSCLC are essential defense mechanisms against tumor progression.

Immune-inhibitory pathways, immune checkpoints, contribute to regulating normal immune responses and maintaining immune tolerance. Some tumor cells exhibit the ability to misuse the immune checkpoint pathways to protect themselves from immune surveillance by blocking the T cells activity through immune checkpoint ligands expressed on the tumor cell surface including programmed cell death ligand 1 (PD-L1), programmed cell death ligand 2 (PD-L2), B7-1, B7-2, liver and lymph node sinusoidal endothelial cell C-type lectin (LSECtin/CLEC4G), Fibrinogen-like protein 1 (FGL1), Galectin-3, HMGB1, PtdSer, Cecam2, Galectin-9, CD112/CD155, and some other not fully characterized. The best characterized and clinically tested immune checkpoint is programmed cell death protein 1 (PD-1)/PD-L1 or PD-L2 pair (80–83).

#### The role of PD-1/PD-L1 in immune system suppression

**Figure 2:**
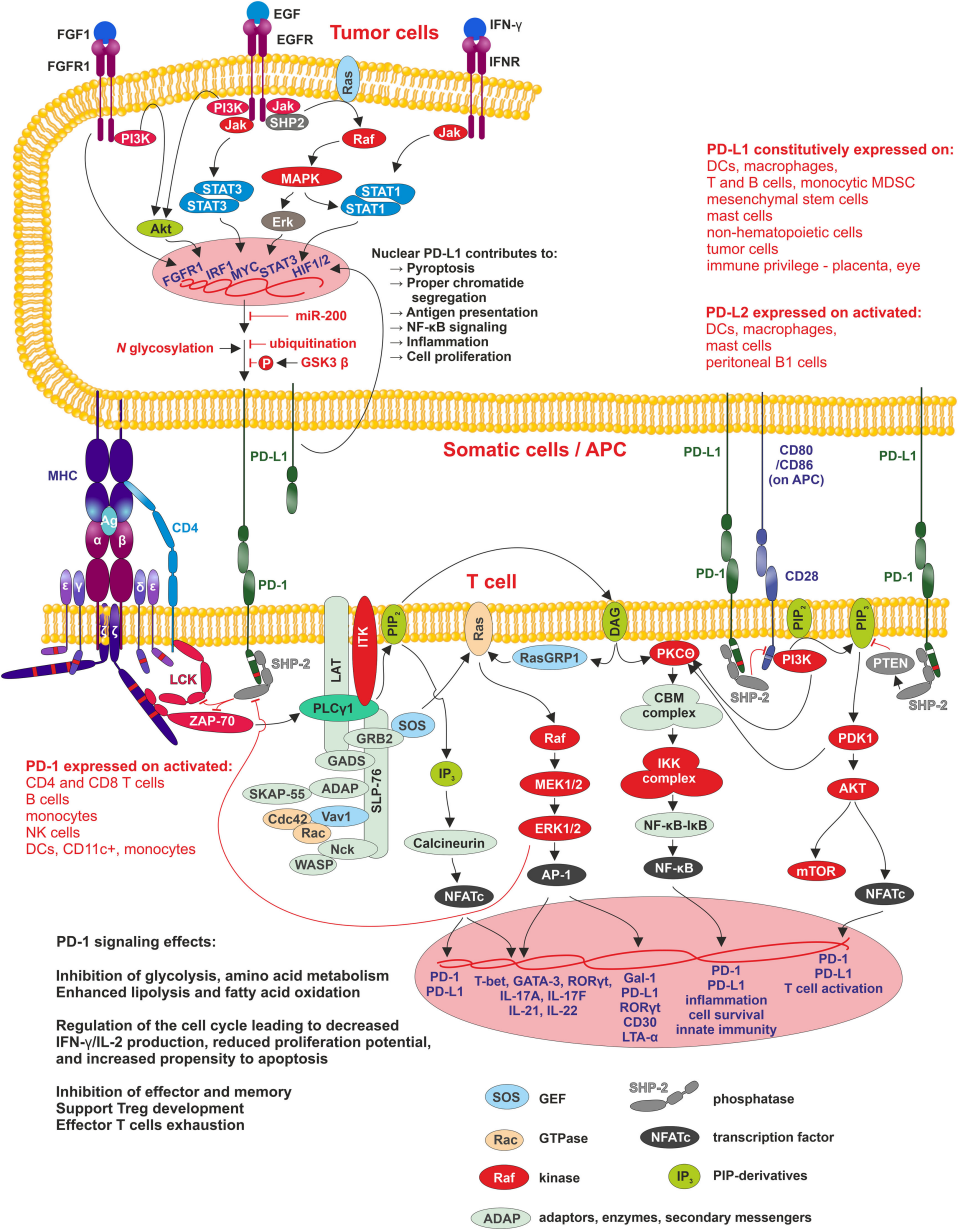
PD1/PD-L1 signaling Interaction of APC, tumor or other cells-expressing PD-L1 (PD-L2) with PD-1 leads to phosphorylation of PD-1 cytoplasmic domain containing two motifs - ITIM-pY223 (white band in PD-1 cytoplasmic domain) and ITSM-pY248 (red band in PD-1 cytoplasmic domain), of which ITSM is crucial for activation of SHP2 phosphatase. SHP2 inhibits two pathways involved in the regulation of T cell differentiation, proliferation, and activation. The first pathway is antigen-dependent and is activated by MHC-peptide-TCR/CD4/8 interaction where SHP2 suppress Lck and ZAP70 kinases signaling through PLCγ to three pathways MAPK/ERK/AP-1, PIP3/IP_3_/Calcineurin/NFATc, and PIP3/DAG/PKCΘ/NF-κB. The second pathway, PI3K/Akt, is antigen-independent, and here SHP-2 suppresses PIP_2_ phosphorylation. In tumor cells, PD-L1 expression positively correlates with the activity of MAPK/ERK signaling pathway, where SHP-2 is its positive regulator. SHP-2 binds to autophosphorylated growth factor receptors, Grb2-associated binder proteins, and signal regulatory protein α that are tyrosine-phosphorylated. Mutations in SHP-2 and other proteins in this signaling pathway, found in many different cancers, are connected with increased MAPK/ERK activity resulting in increased PD-L1 expression and thus tumor cell-controlled suppression of surrounding immune cells within TME and finally, they support tumorigenesis. Membrane-bound PD-L1 can be modified by HDAC-2 and transferred into the nucleus, where promotes transcription of genes involved in antigen presentation, NF-κB signaling, immune checkpoints, inflammation, proper chromatid segregation and pyroptosis.

PD-1/PD-L1 signaling has an important function in immune tolerance due to its ability to suppress the immune response. Its impairment leads to the induction of autoimmunity, progression of chronic infections and tumor growth (**Figure 2**).

**Figure 3:**
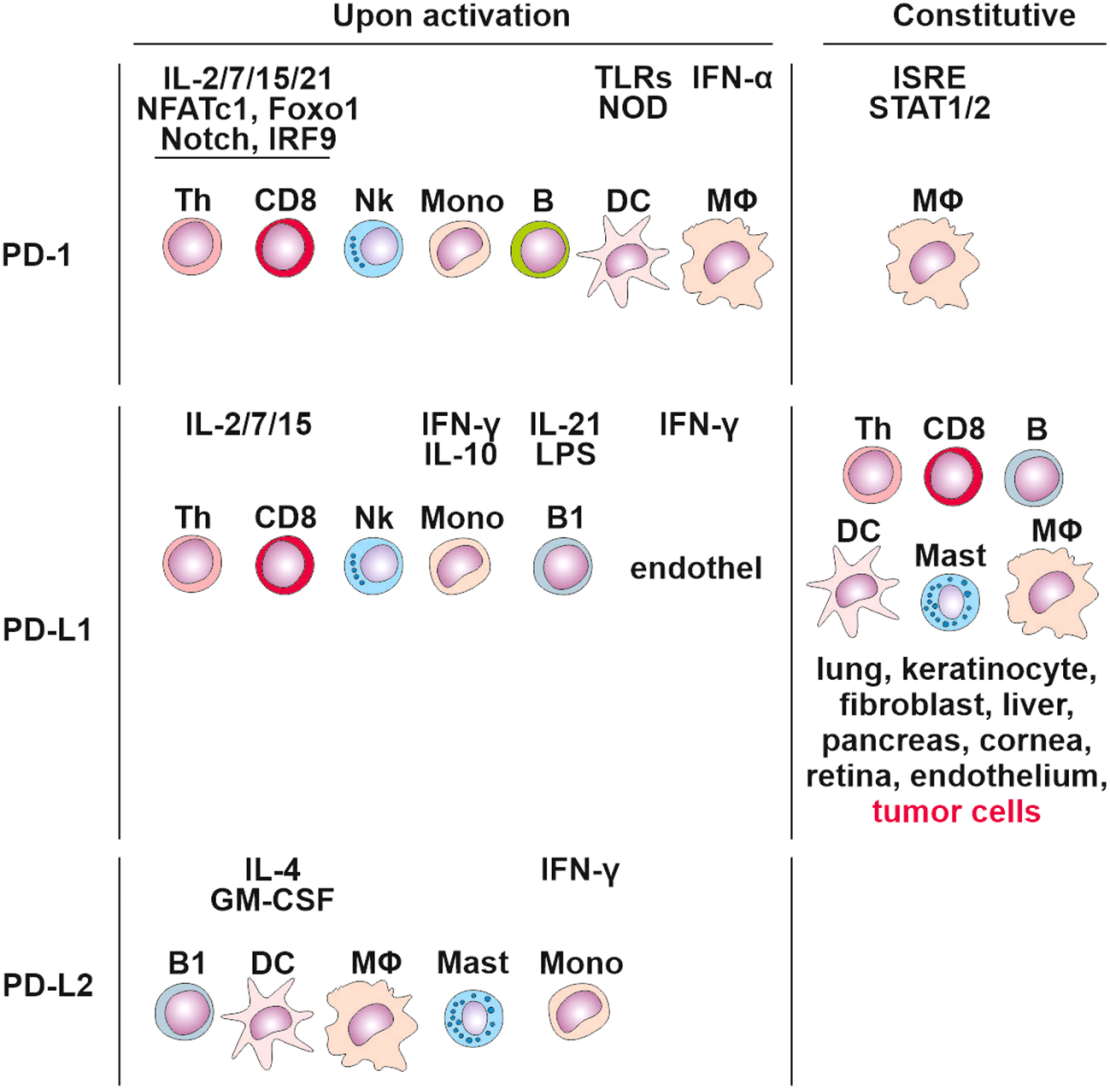
Cells expressing PD-1, PD-L1 and PD-L2 constitutively and upon activation. Tertiary lymphoid structures.

PD-1 signaling affects both the adaptive and innate immune responses. Its expression was originally linked to the regulation of activity of T cells, B cells, DCs, macrophages, monocytes, and NK cells (80, 84) (**Figure 3**), and is regulated by 10 transcription factors: eight activators (c-fos/AP-1, STAT3, STAT4, ISGF3, FoxO1, Notch, NFATc1, and NF-κB) and two inhibitors (Blimp-1 and T-bet) (85). Populations of developing thymocytes and resting naïve T cells show low basal-level expression of PD-1 (86), which has been linked to immune tolerance (87). During an acute infection or the initial stage of tumor development, the generated pathogen/tumor-derived antigens stimulate the maturation of T lymphocytes towards effector and memory subsets. Effector T cells target the infected/tumor cells and eradicate them. In contrast to acute T cell activation, permanent TCR stimulation promotes T cell exhaustion associated with upregulating surface co-inhibitory receptors such as PD-1, TIM-3 and LAG-3, which prevents the generation of autoreactive T cells.

PD-1 interacts with PD-1 ligands (PD-L1 and PD-L2). PD-L1 is expressed in T cells, B cells, macrophages, DCs, bone marrow-derived mast cells, and tumor cells (88) (**Figure 3**). In contrast, PD-L2 expression is greatly limited, as it is mainly detected on activated macrophages, DCs, and some tumor cells (89). DCs have the potential to express PD‐L1 on their surface (90), and studies on other cancers, including colorectal cancer, have discovered that PD‐L1 positive DCs are better predictors of response to anti-PD‐L1 immunotherapy compared to PD‐L1^+^ tumor cells (91).

Cancer cells utilize the expression of PD-L1 as an “adaptive immune mechanism” to escape from the immune anti-tumor responses (80). 55% of NSCLC patients are positive for PD-L1 (92). The expression of PD-L1 is typically linked to the characteristics of the immune microenvironment enriched with CD8^+^ T cells, generation of Th1 cytokines, chemical factors, interferons and specific gene expression (93). PD-L1 expression, therefore, can be categorized as constitutive and inducible based on the extrinsic or intrinsic stimuli. The tumor cells exhibit constitutive PD-L1 expression mediated by dysregulation of tumor suppressor genes or oncogenic signaling pathways. For example, activation of the PI3K/AKT signaling pathway can enhance the expression of PD-L1 via mediating an increase in extrinsic signaling or a decrease in negative regulators, like PTEN. The downregulation of PTEN has been suggested to activate the PI3K/AKT which further accelerates the PD-L1 expression (94). The other signaling pathway involved in the upregulation of PD-L1 is the MAPK signaling pathway (95), and recently the JAK/STAT pathway has also been stated to produce PD-L1 expression in cancers. Additionally, fibroblast growth factor receptor 2 (FGFR2) signaling was also documented to substantially activate the JAK/STAT3 signaling pathway, which in turn caused enhanced expression of PD-L1. Altogether, PI3K/AKT, MAPK, and JAK/STAT3 pathways lead to overexpression of Myc, an oncogenic transcription factor, which binds to the PD-L1 promoter and enhances its expression. Increased Myc is observed in ∼70% of tumors (96, 97). Moreover, genetic, or pharmacological deactivation of Myc has been reported to attenuate the mRNA expression of PD-L1 levels and regenerate the anti-tumor responses in the TME (98). PD-L1 expression, on the other hand, can also be upregulated by DNA double-strand breaks which trigger the STAT signaling pathway via ataxia-telangiectasia mutated (ATM) and Rad3-related checkpoint kinase 1 (Chk1) (99). Other genetic drivers contributing to the high expression of PD-L1 are the mutations in ALK, the HIF1/2α and nuclear factor κB (NF-κB) (96). The tumor-immune cells microenvironment augments PD-L1 expression with pro-inflammatory cytokines, such as IFN-γ, tumor necrosis factor α (TNF-α), IL-10, interleukin-1 (IL-1) and interleukin-6 (IL-6) (100). IFN-γ regulates PD-L1 expression preferentially on immune cells, whereas PD-L1 expression on tumor cells is epigenetically regulated and associated with hypomethylation of PD-L1 promoter (101). In some NSCLC cases, PD-L1 amplification of the genomic region on chromosome 9p24.1 appeared and led to enhanced PD-L1 expression (102). The role of PD-L1 in cellular membrane of tumor cells is well known. Currently, the role of PD-L1 in nucleus of tumor cells were described. Hypoxia present in rapidly growing tumor tissue induce histone deacetylase 2 (HDAC2)-dependent deacetylation of membrane PD-L1, and promotes its nuclear translocation. Nuclear PD-L1 can evade conventional anti-PD1/PD-L1 therapies and can effectively promote tumorigenesis by regulation of angiogenesis by EGR1 regulation (103) (**Figure 2**).

The level of PD-L1 is tightly connected with its stability. The fully glycosylated form of PD-L1 was estimated to have a half-life of 12 h, whereas non-glycosylated PD-L1 showed rapid proteolysis with a half-life of 4 h. Furthermore, decorated glycans were predicted to protect PD-L1 against proteasome degradation, and thus improving its molecular contacts with PD-1 on CD8^+^ T cells (104). In contrast, glycosylation of PD-1 has been a topic of discussion as some studies claim the highly glycosylated PD-1 protein is essentially required in PD-1/PD-L1 interaction (105) while others deciphered the distant location of glycosylation sites from the PD-L1 binding pocket on PD-1 and suggested no direct role of glycosylation in PD-1/PD-L1 interaction (106). Studies describing glycosylation level of PD-L1 in NSCLC tumor cells still need to be completed. We hypothesize that NSCLC tumor cells not only constitutively express PD-L1, but also exhibit a high level of glycosylation in comparison with other tumors.

Higher levels of both PD-L1 and IDO proteins in tumor tissue are independent negative prognostic factors for overall survival in resected NSCLC patients. Immunosuppressive microenvironment - exhausted T-cells together with a decrease in tryptophan, promotes tumor growth, increases the risk of progression and unfavorable prognosis (107).

#### Other molecules involved in immune system suppression - CTLA-4, TIM-3, LAG-3

CTLA-4 (cluster of differentiation 152, CD152) has been identified as the surface receptor of activated T cells and is constitutively expressed on Treg cells, where constitutive expression of CTLA-4 is controlled by FoxP3 (108, 109), and essentially contributes to the regulation of T cell homeostasis and self-tolerance. The current study of Hiroshi Saijo shows that anti-PD-1 antibody monotherapy might be less effective against large NSCLC due to the infiltration of Treg cells. Therefore, it might be appropriate for large NSCLC to select a treatment including an anti-CTLA-4 antibody, which can target Treg cells (110). In naïve T cells, CTLA-4 is primarily located in the cytoplasm since it is exposed to constitutive endocytosis from the plasma membrane, which results in the internalization of 90% CTLA-4 (111). The functional activity of the CTLA-4 receptor primarily includes competition with CD28 receptors for binding to B7 ligands (B7-1/CD80 and B7-2/CD86) on APC. CTLA-4 and CD28 facilitate opposing functions in T cell activation. In naïve T cells, stimulatory signals resulting from both TCR and CD28:B7 binding induce upregulation of CTLA-4 and its transfer to plasmatic membrane. CTLA-4 is known to interact with B7 ligands with higher affinity and avidity than CD28 (111, 112). The functional role of CTLA-4 is to inhibit the priming, activation and migration of T cells (111). In the case of Treg-expressed CTLA-4 interaction with APC-expressed CD80/CD86 induces the upregulation and secretion of indoleamine 2,3-dioxygenase by APC, contributing to the creation of an extrinsic immunosuppressive environment. CTLA-4/B7 function in different phases in comparison to the PD-1/PD-L1 axis, and thus synergistic inhibition of both signaling pathways offers a potential solution to reduce tumor resistance during ICI monotherapy (113, 114). Although CTLA4 expression was found in multiple tumor cells including NSCLC, its function in these cells is largely unknown and is correlated with poor prognosis (115). In NSCLC, CTLA-4 positivity was identified on tumor epithelial cells as well as on tumor stromal compartments with relatively homogenous distribution. The prognostic impact of CTLA-4 on tumor epithelial cells is favorable, since 5-year OS is 29% in CTLA-4 positive vs 19% in CTLA-4 negative primary tumors (115).

T cell immunoglobulin and mucin domain 3 (TIM-3) is a member of the TIM family of genes known for their expression on T cells in allergy and autoimmune diseases. It was initially identified as a specific cell surface marker of interferon (IFN-γ)-producing Th1 and CTL cells (116, 117). TIM-3 has been studied as a negative regulator which essentially contributes to immune tolerance. For instance, upregulated TIM-3 expression is noticed in exhausted CD8^+^ T cells in chronic infections and tumors, while its expression on CD4^+^ T cells is correlated with poor prognosis of NSCLC (118). Tumor cells could inhibit T cells by surface expression or by releasing several TIM-3 ligands such as PtdSer, HMGB1, or Galectin-9 (119). In their study, Zhuang et al., 2012 show TIM-3 positively stained on 92.9% of TILs and most infiltrated tumor-associated macrophages (TAMs) in lung cancer tissues, and 87% of NSCLC primary tumors were positive for TIM-3 (120). Another, more comprehensive study shows TIM-3 located on 25.3% of TILs in NSCLC tumors (121). There are currently a number of clinical trials investigating the use of TIM-3 blockers in NSCLC. Most of them aim for combination of two targets - TIM-3 and PD-1 (e.g. NCT06162572, NCT03708328, NCT04931654, source clinicaltrials.gov).

LAG-3 is an immune inhibitory receptor that is expressed on the surface of activated T cells, pDC, NK cells and B cells. Important roles of LAG-3 are inhibiting Th1 cell proliferation and reducing IL-2, IFN-γ, and TNF secretions (113). The presence of LAG-3 on TILs in tumor tissues has been linked to the poor prognosis of NSCLC (122, 123). LAG-3 is established to regulate the T cell-induced immune response in three ways: (i) directly by stopping the T cells activation and proliferation through negative regulation of T cells, (ii) by enhancing the inhibitory activities of Tregs, and (iii) by preventing the activation of T cells via regulation of APCs (124). LAG-3 ligands such as MHC II, Galectin-3, LSECtin, and FGL1 are expressed on DCs and some other cells. Importantly, the expression of FGL1 was confirmed on lung adenocarcinoma cells (125) which indicates another mechanism of tumor-mediated immune cell suppression. LAG3 is strongly associated with proliferation in NSCLC and significant LAG3 co-expression with other ICI targets suggests its plausible use in clinical trial selection and patient stratification for combination immunotherapy strategies (126). LAG-3 is often connected with poor prognosis (123).

In newly diagnosed NSCLC patients, PD-1, LAG-3 and TIM-3 were detected in TILs from 55%, 41.5%, and 25.3%, respectively. Primarily, PD-1 and LAG-3 were localized on T cell subsets while higher expression of TIM-3 was noticed in macrophages and NK cells. Co-expression of PD-1, LAG-3, and TIM-3 has been related to the activation of T cells, proliferation, and higher expression of proapoptotic markers (FAS/BIM) (121). Thus, combination therapy is a promising target, which is already present in many clinical studies (ClinicalTrials.gov).

OX40, a member of the TNFR family, is involved in the activation of anti-tumor immune response. Its expression is induced on the T cell surface after antigen recognition and coincides with the expression of OX40 ligand (OX40L) mainly on the activated B cells, APCs, NK cells and macrophage cells (113, 127). Activation of OX40 can enhance Th1/Th2-mediated antitumor immune response and, conversely, suppress the suppressive function of Treg cells (127). Enhanced OX40 signaling induced by OX40 agonist antibodies has been observed to augment the T cell-mediated anti-tumor immunity and support enhanced T cell infiltration into tumors, which further results in tumor regression and prolonged survival. Biological activity and the role of the soluble form of OX40 and OX40L in patients with a solid tumor are not well investigated, however, the elevated levels of OX40 and OX40L in serum can be linked with poor prognosis and may display the immune-exhausted status against lung adenocarcinoma (128). In NSCLC, high OX-40 expression in the tumor immune infiltrate is associated with a favorable prognosis. Its prognostic utility is independent of PD-L1 and other common markers of immune activation. High OX-40 expression potentially identifies a unique subgroup of NSCLC that may benefit from co-stimulation with OX-40 agonist antibodies and potentially enhance the efficacy of existing immune checkpoint therapies (129).

TIGIT is an inhibitory immune receptor from the poliovirus receptor (PVR) family of immunoglobulin proteins (113). The biological role of TIGIT involves interactions with the CD155 expressed on APCs or tumor cells to down-regulate the functions of NK cells and T cells (130). Like in other types of cancers, enhanced expression of TIGIT was observed in NSCLC and can be associated with increased levels of other immune inhibitory receptors, including PD-1, LAG-3, TIM-2. We can expect that dual inhibition of immune receptors can offer a favorable immunotherapy. As a mice experiment has already shown, the positive effect of dual blockade TIGIT and PD-1/PD-L1 resulted in complete tumor rejection and overall prolonged survival (131). In NSCLC, the prognostic value of TIGIT is still questionable, however, high TIGIT density correlates with advanced TNM score (132). Blocking TIGIT in NSCLC requires further investigation, but TIGIT will likely belong to promissing ICI targets in combination therapy.

### Peripheral blood markers in NSCLC diagnosis

Histopathological assessment is the gold standard for diagnosing NSCLC. However, due to its invasive nature, testing for tumor markers in the peripheral blood is more convenient for the patient. Such blood markers can be utilized as a suitable tool for possible characterization of pathological types of NSCLC and applied therapy. In the clinical diagnosis of NSCLC, the presence of cytokeratin 19 fragments antigen (CYFRA21-1), carcinoembryonic antigen (CEA) and squamous cell carcinoma antigen (SCC-Ag) in the peripheral blood are used as reference tumor biomarkers. CYFRA21-1 in the blood is more frequent in NSCLC compared to other cancers (133). Additionally, other common tumor blood markers increased in NSCLC are carbohydrate antigen 125 (CA125), CA15-3, CA72-4 and CA19-9.

Patients with longer overall survival showed elevated levels of CD3^+^, CD4^+^ and CD8^+^ T cells, while peripheral blood NK cell counts were reduced (134). Moreover, such patients also showed a statistically significantly reduced PD-1 expression on both CD3^+^ and CD8^+^ T cells. The prognostic value of PD-1 expression on CD4^+^ T cells in the peripheral blood of patients with NSCLC is evident and shows that high PD-1 expression is associated with poor clinical outcomes (135). Testing peripheral expression of PD-1 can potentially calculate a clinical response to the given therapy based on the inhibition of the PD-1/PD-L1 axis (136). Notably, a positive correlation between blood PD-L1 and tumor PD-L1 expression has been established and could serve as a positive biomarker of efficacy and overall survival in advanced NSCLC patients (137). Another proficient marker is the presence of responsive memory T cells against TAAs in the peripheral blood of NSCLC patients (46).

Low-density neutrophils (LDNs) present in peripheral blood are increased in NSCLC and marked as potential novel biomarkers for the characterization of NSCLC tumors. LDNs, comprised of subsets of both immature and mature neutrophils, have been linked with immunosuppressive functions as opposed to the high-density neutrophils (HDNs) which showed anti-tumorigenic properties (138).

Several studies have attempted to decipher the molecular biomarkers or prognostic factors from the peripheral blood to identify the chances of lung cancer metastasis or recurrence. In this context, efforts are made to identify potential endothelial, hematological, or inflammatory markers in NSCLC patients. Recent studies have identified increased levels of RANTES, sVCAM-1, sE-selectin, HMGB1, Ang-2, VEGF, PAI-1 and platelet-derived microparticles (PDMP) in NSCLC patients (139). Not only PDMP, but also mean platelet volume and platelet counts are important prognostic factors in NSCLC (140–142).

Initially, immune checkpoints were expected to be expressed only on cell membranes. However, recent research confirmed that they are also present in soluble forms (s) in tissue fluids including serum as exemplified for sTIM-3, s4-1BB, sCD27 (sTNFRSF7), sLAG-3, sIDO, sPD-L2, sCTLA-4 (sCD152), sB7-1 (sCD80) and sPD-1 (143). Although not characterized in detail, soluble forms of immune checkpoints have been reported to play a critical role in immune regulation in a variety of tumors and inflammatory conditions. They are biologically active and dependent on membrane function in either positive or negative regulation in the paracrine and systemic manner. For example, sPD-1 may function as an immune stimulator, in contrast to membrane-bound PD-1, whereas sPD-L1 retains suppressive activity (144). sTIM-3 could promote tumor growth and inhibit T cell proliferation as well as IL-2 and IFN-γ secretion. In a cohort of NCSCLC patients, serum levels of sTIM-3, sCD137, sCD27, sLAG-3, sIDO, sPD-L2 and sCTLA-4 were significantly higher when compared to the control group. Furthermore, sTIM-3, cCD137 and sCD27 exhibit significantly higher serum levels in advanced NSCLC when compared to early stages (143). In addition, TIM-3 and LAG-3 were identified as independent biomarkers for the early diagnosis of NSCLC and the combination of TIM-3, LAG-3 and CD137 could increase the diagnostic accuracy (143, 145).

In addition to secreted forms of immune checkpoints, exosomes could contain membrane-bound immune checkpoints exhibiting important immunosuppressive roles. In NSCLC, exosomal PD-L1 could suppress the secretion of IL-2 and IFN-γ, leading to apoptosis of CD8^+^ T cells (146). In experimental mice, injection of tumor cells that were unable to produce PD-L1-decorated exosomes induced a memory and effector immune response that prevented the growth of wild-type tumor cells administered to a specific part of the mouse’s body, even several weeks later (147). The same report documented that tumor-generated exosomal PD-L1 acts systemically, including draining lymph nodes where it inhibits T cell activation. Therefore, inhibition of generating PD-L1 decorated exosomes represents a new way of tumor therapy. It could be proposed that other immune checkpoint molecules could systemically circulate with exosomes and microsomes, urging the need for further research.

In addition to immune suppression mediated by binding PD-L1 to T cell surface-exposed PD-1, it was reported that PD-L1 could be actively transported into the nucleus of tumor cells, where it could affect cell division and genomic instability by affecting sister chromatin cohesion and immune evasion by affecting transcription of many immune genes, MHC presentation, and pyroptosis as detailed in review by Xiong et al. (148). Nuclear localization of PD-L1 was associated with shorter survival in colorectal cancer (149).

Another potential marker to predict disease progression in NSCLC is small non-coding RNAs (miRNAs). There are currently seven miRNAs (miR-215-5p, miR-411-3p, miR-493-5p, miR-494-3p, miR-495-3p, miR-548j-5p and miR-93-3p) and their presence in the patient serum helps to distinguish good responders from poor responders to nivolumab (150).

Recently, long noncoding RNAs (lncRNAs) have been suggested as potential clinical indicators due to their stability and tissue specificity and their detection in various body fluids (151). lncRNAs can be used as markers to distinguish early-stage disease from healthy patients with high sensitivity and specificity and to provide prognostic insight into the risk of metastases and recurrence (152).

## Current and future directions of immune checkpoint inhibitors therapy

### Antibodies – current tool for NSCLC treatment

There are currently seven monoclonal antibodies approved for the treatment of NSCLC. Three of them (Pembrolizumab, Nivolumab, Cemiplimab) target PD-1, the other two (Tremelimumab, Ipilimumab) target CTLA-4 and two others (Atezolizumab and Durvalumab) are anti-PD-L1 mAbs (**Table 1**).

**Table 1:** FDA/ EMA proved antibodies targeting immune checkpoints.

Target	FDA/EMA approved	Year of FDA/EMA approval	Name	Brand Name	Type	Disease
PD-1	yes	2017	Pembrolizumab	Keytruda	humanized monoclonal IgG4 kappa antibody	NSCLC Urothelial carcinoma Melanoma Hodgkin lymphoma Head and neck squamous cell carcinoma Cervical cancer Gastric cancer Metastatic colorectal cancer
		2016	Nivolumab	Opdivo	humanized IgG4 monoclonal antibody	NSCLC SCLC Urothelial carcinoma Melanoma Renal cell carcinoma Lung cancer Hodgkin lymphoma Metastatic colorectal cancer
		2018	Cemiplimab	Libtayo	recombinant human IgG4 monoclonal antibody	Cutaneous squamous cell carcinoma
PD-L1	yes	2016	Atezolizumab	Tecentriq	Fc-engineered, humanized, non-glycosylated IgG1 kappa immunoglobulin	NSCLC SCLC Triple negative breast cancer Urothelial carcinoma
		2017	Avelumab	Bavencio	human IgG1 lambda monoclonal antibody	Merkel cell carcinoma Urothelial carcinoma Renal cell carcinoma
		2017	Durvalumab	Imfinzi	human immunoglobulin G1 kappa (IgG1κ) monoclonal antibody	NSCLC SCLC Urothelial carcinoma Hepatocelular carcinoma
CTLA-4	yes	2020	Ipilimumab	Yervoy	recombinant IgG1 kappa immunoglobulin	NSCLC Melanoma Renal cell carcinoma Mesothelioma Metastatic colorectal cancer Mesothelioma Esophageal cancer
		2022	Tremelimumab	Imjudo	human IgG2 monoclonal antibody	NSCLC Hepatocellular carcinoma
LAG-3	yes	2022	Relatlimab	Opdualag	human IgG4 monoclonal antibody	Melanoma

#### Anti-PD1 antibodies

Pembrolizumab (Keytruda, Merck Sharp & Dohme) is a humanized monoclonal IgG4 antibody against the immune checkpoint protein PD-1. Pembrolizumab was first approved in the United States (US) by the Food and Drug Administration (the FDA) in September, 2014 and later in Europe by the European Medicines Agency (EMA) in July, 2015. In, 2019, the FDA and EMA approved Keytruda as first-line treatment in metastatic NSCLC where tumor proportion score (TPS) is greater than 50% (82) and second-line treatment for patients with PD-L1 expression TPS exceeding 1%. For evaluation of the PD-L1 TPS in NSCLC tissue samples, the FDA approved a validation assay from Dako/Agilent for qualitative assessment of PD-L1 protein expression in formalin-fixed paraffin-embedded (FFPE) tissue samples of NSCLC patients. This assay utilizes monoclonal mouse anti-PD-L1 antibody clone 22C3, designed for immunohistochemical (IHC) analysis. In, 2018, the FDA approved Keytruda in combination with pemetrexed and platinum chemotherapy as the first-line treatment of patients with metastatic nonsquamous NSCLC, with no EGFR or ALK genomic tumor aberrations, and in the same year, in combination with carboplatin and either paclitaxel or paclitaxel protein-bound, as first-line treatment of patients with metastatic squamous NSCLC (**Table 2**).

**Table 2:** Profiling of NSCLC patients to FDA/EMA approved therapies.

Target	Drug	Age	Type of tumor	Type of treatment	Determination of target positivity	Presence of EGFR and ALK mutations	Combination with other chemotherapy	OS (years) % of survival (Drug vs other)
PD-L1	Keytruda	FDA – not determined EMA – age 12 and older	metastatic non-squamous	First line treatment	no	no	pemetrexed and platinum chemotherapy	KEYTRUDA + platina+pemetrexed vs platina+pemetrexed 5 years 19,4 % vs 11,3 %
			metastatic squamous	First line treatment	no	Not determined	carboplatin and either paclitaxel or paclitaxel protein-bound	KEYTRUDA + carbo + paclitaxel vs carbo + paclitaxel 5 years 18,4 % vs 9,7 %
			Nonsquamous and Squamous Advanced	First line treatment	TPS ≥1%	no	Single agent therapy	KEYTRUDA vs Platinum-containing chemotherapy 5 years 32 % vs 16 %
			metastatic		TPS ≥1%	Yes, before Keytruda treatment	Single agent therapy – disease progression on or after platinum-containing chemotherapy	
PD-1	Opdivo	FDA- unspecified or adult dependent on therapy EMA – not recommended under age 18 and older	resectable	Neoadjuvant treatment	no	Not determined	platinum-doublet chemotherapy	Opdivo + chemo vs chemo 2 years 83 % vs 71 %
			metastatic	First line treatment	TPS ≥1%	no	ipilimumab	Yervoy + Opdivo vs chemo 5 years 24 % vs 14 %
			metastatic or recurrent		no	no	ipilimumab and 2 cycles of platinum-doublet chemotherapy	Yervoy + Opdivo + chemo vs chemo 3 years 25 % vs 15 %
			metastatic with progression		no	yes	Platinum based progression	Opdivo + chemo vs chemo 5 years 13,4 % vs 2,6 %
PD-L1	Tecentriq	FDA – adult EMA - adult	stage II to IIIA	adjuvant treatment following resection	TPS ≥1%	Not determined	Single agent therapy following platinum based chemotherapy	Tecentriq vs best supportive care 4 years 79,3 % vs 70,9 %
			metastatic	First line treatment	TPS ≥50% and presence of IC ≥10%	no	Single agent therapy	
			metastatic non-squamou	First line treatment	no	no	bevacizumab, paclitaxel, and carboplatin,	
			metastatic non-squamou	First line treatment	no	no	paclitaxel protein-bound and carboplatin	
			metastatic		no	Yes with disease progression	Single agent	
PD-L1	Imfinzi	FDA – adult EMA - adult	unresectable Stage III whose disease has not progressed following concurrent platinum-based chemotherapy and radiation therapy		no	Not determined		Imfinzi vs chemoradiotherapy 5 years 43 % vs 33 %
CTLA-4	Yervoy	FDA – adult EMA - adult	metastaic	First line treatment	TPS ≥1%	no	nivolumab	Yervoy + Opdivo vs chemo 5 years 24 % vs 14 %
			metastatic or recurrent	First line treatment	no	no	Nivolumab and 2 cycles of platinum-doublet chemotherapy	Yervoy + Opdivo + chemo vs chemo 3 years 25 % vs 15 %
CTLA-4	Imjudo	FDA – adult EMA - adult	metastaic		no	no	durvalumab and platinum-based chemotherapy	Imjudo + Imfinzi vs Sorafenib) 3 years 30 % vs 20 %

Nivolumab (Opdivo, Bristol-Myers Squibb Company), IgG4 anti-PD-1 monoclonal antibody was approved by the FDA and EMA (2015) for treatment of both squamous and non-squamous NSCLC, and as second-line therapy in patients with metastatic NSCLC (153). In, 2020, nivolumab was approved in combination with Ipilimumab as first-line treatment for patients with metastatic NSCLC whose tumor cells express PD-L1≥1%. On March 4, 2022, the FDA and EMA approved nivolumab with platinum-doublet chemotherapy in adult patients with resectable NSCLC in the neoadjuvant setting (**Table 2**).

Cemiplimab (Libtayo, Regeneron Pharmaceuticals, Inc., and Sanofi) is a monoclonal IgG4 antibody against PD-1. The FDA and EMA approved Libtayo monotherapy (if PD-L1 expression is ≥ 50% of tumor cells) or in combination with chemotherapy (PD-L1 1-49%) as first-line therapy for adult NSCLC patients who express PD-L1 (in ≥50% tumor cells), with no EGFR, ALK or ROS1 aberrations, and who have locally advanced or metastatic NSCLC and are not candidates for definitive chemoradiation therapy.

Unfortunately, PD-1 inhibitor treatment can cause severe side effects of immune origin - immune-related adverse events (irAEs), such as hepatitis, colitis, and skin disorders. However, irAEs have been reported to be associated with good clinical outcomes while causing treatment discontinuation and death (154–157).

#### Anti-PD-L1 antibodies

Atezolizumab (Tecentriq, Genentech, Inc.) is a monoclonal human IgG1 antibody. In, 2016, the FDA approved Atezolizumab as a therapy for patients with metastatic NSCLC who have experienced disease progression during or following platinum-containing chemotherapy, and if their tumor has EGFR or ALK gene abnormalities. Tecentriq is currently used in combination with Avastin (bevacizumab), paclitaxel and carboplatin (chemotherapy), for the initial (first-line) treatment of individuals with metastatic NSCLC with no EGFR or ALK genomic tumor aberrations. Additionally, it is used as monotherapy for first-line treatment of adult patients with metastatic NSCLC whose tumors have a PD-L1 expression in ≥ 50% tumor cells or ≥ 10% tumor-infiltrating immune cells and lacks EGFR or ALK genomic tumor aberrations. In, 2021, the FDA approved Tencentriq as an adjuvant treatment for patients with stage II to IIIA NSCLC whose tumors have PD-L1 expression on ≥ 1% of tumor cells, as determined by an FDA-approved VENTANA PD-L1 (SP263) assay (Ventana Medical Systems, Inc.), following resection and platinum-based chemotherapy.

Durvalumab (Imfinzi, AstraZeneca Inc.) is a monoclonal anti-PD-L1 IgG1 kappa antibody that was approved in, 2018 by the FDA and EMA for treating patients with stage III NSCLC whose tumors cannot be surgically removed and whose cancer has not progressed after chemotherapy and radiation treatment. Nowadays, 141 clinical trials are testing Durvulumab in other stages of NSCLC individually or in combination with other drugs (source:ClinicalTrials.gov) (158).

Avelumab (Bavencio., EMD Serono, Inc. and Pfizer, Inc.) is a human monoclonal IgG1 antibody, which has been approved for the treatment of metastatic Merkel cell carcinoma and is used in therapy against advanced urothelial carcinoma. Currently, 10 ongoing clinical trials are testing Avelumab in NSCLC patients (source: ClinicalTrials.gov). To evaluate PD-L1, Dako PD-L1 IHC 22C3 pharmDx assay and Dako PD-L1 IHC 73-10 assay are used as quantitative evaluators (159).

The effectiveness of anti-PD-1/PD-L1 treatment is tightly connected with glycosylation of PD-1/PD-L1, which is crucial for proper PD-1 and PD-L1 binding. Therapeutic antibodies such as Avelumab, Atezolizumab and Durvalumab recognize the glycosylated form of PD-L1 in contrast to diagnostic antibodies which favor the non-glycosylated form of PD-L1 (160). Most commercially available antibodies are produced to recognize antigens or recombinant proteins that are expressed in bacterial or other hosts; however, this does not provide the proper glycosylation present in mammalian cells. Removing the glycosylation moieties may help to overcome the steric hindrance and enable more accurate stratification of patients. Lee et al. have demonstrated that the diagnostic antibody clone -8, which is widely used to detect PD-L1 expression and to evaluate formalin-fixed paraffin-embedded (FFPE) tissue samples, has limited recognition of heavily glycosylated PD-L1 forms, which may result in false negative scoring of patients (161). PNGase-treated lung cells showed increased immunofluorescence signal (detected by ELISA assay) compared to untreated samples. In 14% of FFPE tissue samples, PD-L1 TPS was increased from 1% to more than 49% after deglycosylation. This may indicate that certain evaluations using IHC 28-8 may bring false-negative results. Thus, selecting the appropriate PD-L1 diagnostic strategy may affect the therapeutic strategy for the patients and their chances of survival (161).

#### Anti-CTLA-4 antibodies

Ipilimumab (Yervoy, Bristol-Myers Squibb Company) is a human CTLA-4-blocking antibody approved by the FDA and EMA in, 2020. It is used in combination with nivolumab and 2 cycles of platinum-based chemotherapy for first-line treatment of metastatic NSCLC in adults whose tumors express PD-L1 (≥1%) and have no EGFR and ALK mutations (ClinicalTrials.gov Identifier: NCT02658890).

Tremelimumab (Imjudo, AstraZeneca, Inc.) is a human monoclonal antibody, approved by the FDA and EMA, that blocks the activity of CTLA-4 and thus activates the anti-tumor immune response. In September, 2021, AstraZeneca showed that combinatory treatment of Imfinzi (Durvalumab) and Tremelimumab when added to platinum-based chemotherapy, improved overall survival (OS) and progression-free survival compared to chemotherapy alone in first-line treatment of patients with metastatic NSCLC (162).

### Antibodies - future directions in antibody therapies in NSCLC

**Figure 4:**
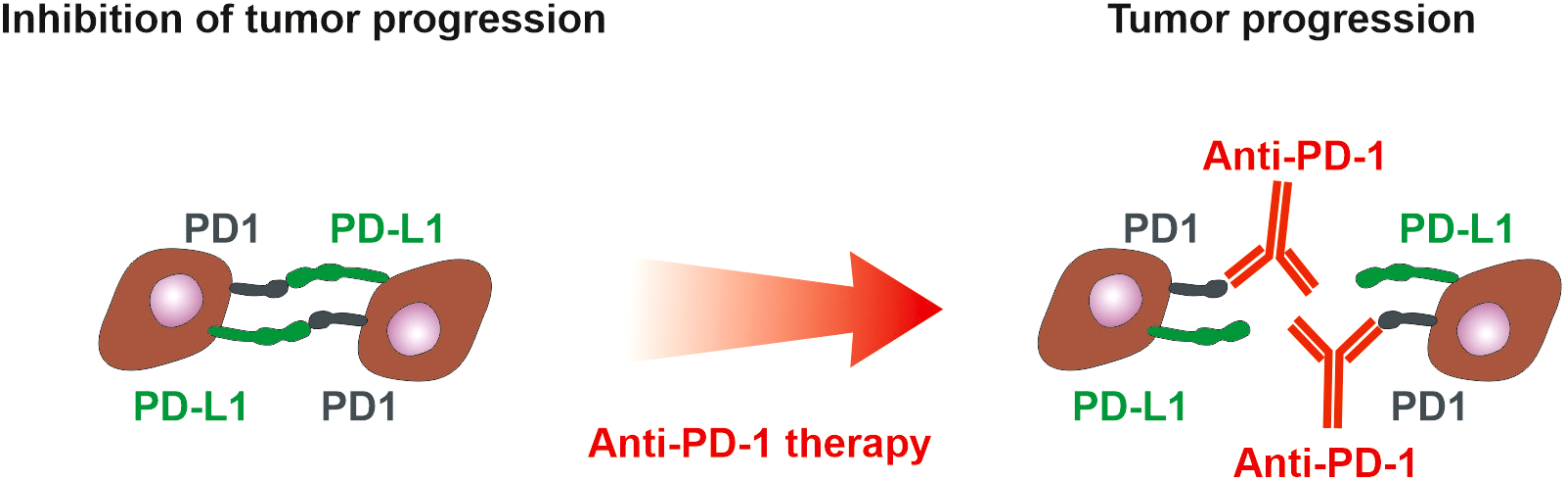
PD-1 and PD-L1 in tumor microenvironment. PD-1 is expressed on immune cells, whereas tumor cells could present both PD-L1 and PD-1. Interaction of PD-L1 on tumor cells with PD-1 on immune cells suppresses immune cells. If tumor cells express both PD-L1 and PD-1, they inhibit each other similarly to the inhibition of immune cells. Anti-PD-1 antibodies block the suppression of immune cells but simultaneously could block the inhibitory action of PD-1 on tumor cells and thus support tumor cell proliferation (165). The anti-PD-L1 and anti-PD1 treatment of NSCLC can exhibit a positive therapeutic effect if the high expression of PD-L1 on tumor cells and/or immune cells is detected (101). However, only 20% of NSCLC patients benefit from anti-PD-1 therapy with progression-free survival of 22 weeks (163), emphasizing the need for assessment of PD-1 on tumor cells.

Standard anti-PD-1/PD-L1 therapy brings lasting benefit only to a certain number of patients (163, 164) (**Figure 4**), therefore a number of combinations of antibody therapy with another type of treatment are currently being developed. Monoclonal antibodies targeting PD-1 or PD-L1 are commonly used in combination with chemotherapy and radiotherapy in treating NSCLC (KEYNOTE-189, Impower130). Other strategies that involve the use of PD-1/PD-L1 inhibitors in combination with other treatments are also being tested in clinical trials. These include the use of telomerase inhibitors. Telomerase activation is a fundamental feature of cellular immortalization and it is considered a crucial step in carcinogenesis. Its activity has been detected in more than 90% of all cancers. The telomerase peptide vaccine UV1 induces Th1 immune response targeting tumor cells and is used with pembrolizumab (anti-PD-1) in a phase-I clinical trial (NCT03538314), with nivolumab (anti-PD-1) and ipilimumab in two randomized phase II clinical trials in malignant melanoma (NCT04382664) and mesothelioma (ClinicalTrials.gov Identifier: NCT04300244), with Durvalumab (anti-PD-L1) and Olaparib (PARP inhibitor) in relapsed ovarian cancer (ClinicalTrials.gov Identifier: NCT04742075) and with Pembrolizumab in head and neck cancer (ClinicalTrials.gov Identifier: NCT05075122). In NSCLC, there are ongoing clinical trials (ClinicalTrials.gov Identifier: NCT02846103, NCT02818426), investigating the effect of UV1 vaccines on NSCLC progression.

There are several clinical studies introducing monoclonal antibody targeting IL-1β Canakinumab (Ilaris, Novartis) into NSCLC treatment. Canakinumab was approved by FDA for the treatment of Periodic Fever Syndromes, active Still’s disease, and gout flares. Nowadays Canakinumab is used in ongoing studies in combination with Durvalumab (ClinicalTrials.gov Identifier: NCT04905316), Pembrolizumab (ClinicalTrials.gov Identifier: NCT03968419), Docetaxel (ClinicalTrials.gov Identifier: NCT03626545), or in combination with cisplatin, carboplatin and Pemetrexed (ClinicalTrials.gov Identifier: NCT03064854).

Since PD-1, PD-L1 monotherapy is insufficient and high co-expression of PD-L1 and IDO proteins was described in NSCLC, several clinical trials proposed dual therapy blocking these proteins in NSCLC are ongoing. There is a clinical trial using the first-in-class immune-modulatory vaccine IO102-IO103 in combination with pembrolizumab (NCT05077709). Vaccine IO102-IO103 activates production of IDO/PD-L1 specific T-cells targeting tumor cells expressing IDO and/or PD-L1. Next clinical trial use combination of an anti-IDO-1 agent (LY3381916) administered alone or in combination with anti-PD-L1 checkpoint antibody (LY3300054) (ClinicalTrials.gov Identifier: NCT03343613), next use combination of Epacadostat (IDO inhibitor) with Pembrolizumab and chemotherapy (ClinicalTrials.gov Identifier: NCT03085914, NCT03322540, NCT03322566), and clinical trial administred combination of BMS-986205 (IDO inhibitor) with Nivolumab and in combination with both Nivolumab and Ipilimumab (ClinicalTrials.gov Identifier: NCT02658890).

TMB is a factor that strongly individualizes each tumor. Different levels of TMB in tumor tissue compared to blood samples could be predictors for development of clinical response. This feature is targeted in clinical trial NCT04392505. Not only TMB, but also epigenetic changes play a key role in the development and progression of cancer. Epigenetic immune modeling of cancer cells is their hallmark, as it impairs functional immune recognition of malignant cells by the immune system. Because of this, there is potential for epigenetic drugs, including DNA hypomethylating agents, to sensitize tumor cells to emerging immunotherapies. Another promising clinical trial is combination of Nivolumab with Ipilimumab and Guadecitabine (hypomethylating agent) or Nivolumab combined with Ipilimumab (ClinicalTrials.gov Identifier: NCT04250246). An ongoing clinical study combining Azacytidine, a hypomethylating drug, Entinostat, inhibiting histone deacetylase with anti-PD-1 therapy – Nivolumab, is under investigation (ClinicalTrials.gov Identifier: NCT01928576) (83, 166). Secondary NSCLC after cancer recurrence with anti-PD1 therapy has much higher promoter methylation compared to primary cancerous or healthy tissue, the combination of Azacytidine, with Entinostat and Nivolumab is very promising.

## Future perspectives on treatment

### Bispecific antibodies

Unlike monospecific antibodies, bispecific antibodies recognize two different epitopes on the same or different antigens. Accordingly, a bispecific antibody must have two different heavy chains and two different light chains (167).

In approximately 70-80% of non-responding patients with melanoma, NSCLC, and renal cell carcinoma, a lack of activated T cells is suggested. Therapies boosting T cell activity may significantly improve their treatment efficacy. Horn et al. have designed bispecific T cell engagers (BiTEs) that activate T cells through CD3 and target activated T cells to tumor-expressed antigen PD-L1 and the BiTEs also activate healthy donor CD4^+^ and CD8^+^ T cells that are specifically cytotoxic for PD-L1^+^ tumor cells (168). Another bispecific antibody (MCLA-145) targets CD137 and PD-L1. The result of its effect is the upregulation of CD137 (on T cells) and PD-L1 (on tumor cells and APCs), the release of pro-inflammatory cytokines and activation of effector T cells targeting tumor cells (169).

### Nanobodies, probodies, affibodies and DARPINs

Nanobodies, probodies, affibodies and DARPINs are small molecules that retain the ability of antibodies to recognize antigens and overcome the size limitations of monoclonal antibodies.

A nanobody is an antibody fragment consisting of a single monomeric variable antibody domain with high solubility, high stability and excellent tissue penetration *in vivo* (170). In addition, the low molecular weight of nanobodies offers the advantage that they can be eliminated by the kidneys, making them highly suitable for treatment due to their low cytotoxicity. For example, Nanobody KN035 (Envafolimab, Tracon Pharmaceuticals) strongly induced T-cell responses and inhibited tumor growth. In terms of effectiveness against tumors, it has been proved to be equal to Durvalumab (171). KN035 has been approved in 10 clinical trials (source ClinicalTrials.gov).

Probodies are therapeutics, produced as an antibody prodrug, that are activated by proteases occurring uniquely in tumors, which limits their activity on TME and minimizes extra-tumor toxicity (172). There are currently no FDA or EMA-approved probodies. The furthest along in development is CX-072 (Pacmilimab), which is a probody immunotherapy that targets PD-L1. An *in vivo* study conducted on mice showed that Pacmilimab accumulated in tumors with only limited uptake in other non-cancerous tissues expressing PD-L1 (173, 174). The clinical trial investigating the suitable dose of CX-072 for patients with solid tumors and lymphomas was completed (ClinicalTrials.gov Identifier: NCT03013491).

Nanobodies and affibodies (originally derived from immunoglobulin binding Z-domain from staphylococcal protein A (175)) can be used as non-blocking nanobody-based radioisotope binding tracers, which possess different binding epitopes compared to monoclonal antibodies. This feature is important during monitoring anti-PD-L1 therapy because non-interference between radiotracer and any anti-PD-L1 therapeutic allows for improving the imaging quality (resolution and quantification) and does not affect the activity of the therapeutic agent (176).

Designed ankyrin repeat proteins (DARPins) are derived from the most common natural protein–protein interaction platform– ankyrin repeats (177). The functionality of DARPins targeting PD-1 has been compared with clinically used anti-PD-1 antibodies, i.e., pembrolizumab and nivolizumab, and found comparable (178). MP0250 is the first DARPin in clinical studies (ClinicalTrials.gov Identifier: NCT03136653, NCT03418532, NCT02194426). It has binding specificities for vascular endothelial growth factor A (VEGF-A), hepatocyte growth factor (HGF) and human serum albumin (HSA). Since MP0250 can increase the efficacy of anti-PD1 therapy in mice, it has the potential for NSCLC treatment (179).

### Antibody-drug conjugates

Antibody-drug conjugates (ADCs) are a prospective class of therapeutic agents for NSCLC treatment. ADCs recognize the specific antigen and then selectively release a highly toxic cytostatic agent or toxin at the tumor site. ADCs already have applications in the treatment of other malignancies, such as HER2-positive breast cancer. Due to the occasional occurrence of HER2 amplifications in NSCLC, the first successful trials using trastuzumab-deruxtecan (Destiny-Lung02) have already been conducted (52). Other molecules are in clinical trials.

### Future direction in immunoscoring for NSCLC diagnostics, prediction and therapy response

Antibodies targeting immune checkpoints increase the OS of NSCLC patients. However, not all patients receiving immunotherapy respond appropriately, break immune suppression and go into remission. This may be connected with insufficient immunoscoring and profiling of cancer patients. Nowadays, immunoscoring of NSCLC patients is individual for different types of NSCLC (**Table 2**). IHC is not required in all stages and types of NSCLC as its positivity is closely connected with appropriate tissue collection and potential tumor heterogeneity. The collected biopsy can be false negative and the patient can be falsely inappropriate for antibody therapy. As tumor development is linked to the presence of serum tumor markers, their justification can be a valuable tool for selecting the appropriate treatment. CEA, CA15.3, SCC, CYFRA 21-1, NSE, and ProGRP are tumor markers assessed in NSCLC patients, however, their presence does not correlate with the efficiency of immune checkpoint therapy. The current research describes new markers and associating them with immune checkpoint deficiency.

Currently, the only available therapy for reactivating the immune system involves antibodies that block the interaction between PD-1/PD-L1 or CTLA-4. The efficiency of this therapy is connected not only with proper patient selection, but also with the size of the antibody and the density of the tumor tissue. Nanobodies, probodies, affibodies and DARPINs, which target immune checkpoints, are very promising tools due to their high binding affinity and small size. Their research is ongoing and some of them are currently in clinical trials.

### The methodology for the literature search and article inclusion criteria

A systematic literature review was conducted using PubMed and Web of Science without publication dates limitation using key search terms including: NSCLC (non-small cell lung cancer), immunity/immune, immune checkpoint, PD-1, PD-L1 and others, returning over 10,000 publications. Focus was placed on clinical studies measuring the efficacy of immune checkpoint therapy in NSCLC using the search through ClinicalTrials.gov database returning 1 555 studies. The authors screened resulting studies independently for relevance.

## Author contributions

LR: Writing – original draft, Writing – review & editing. JM: Writing – original draft. PC: Writing – original draft. PJ: Writing – original draft. OF: Writing – original draft. JŠ: Writing – original draft. PM: Writing – original draft, Writing – review & editing. MR: Writing – original draft, Writing – review & editing.
